# Metformin inhibits esophageal squamous cell carcinoma-induced angiogenesis by suppressing JAK/STAT3 signaling pathway

**DOI:** 10.18632/oncotarget.20341

**Published:** 2017-08-18

**Authors:** Yi Yang, Guoguo Jin, Hangfan Liu, Kangdong Liu, Jimin Zhao, Xinhuan Chen, Dongyu Wang, Ruihua Bai, Xiang Li, Yanan Jang, Jing Lu, Ying Xing, Ziming Dong

**Affiliations:** ^1^ Department of Pathophysiology, School of Basic Medical Sciences, Zhengzhou University, Zhengzhou, Henan 450001, P.R. China; ^2^ Collaborative Innovation Center of Henan Province for Cancer Chemoprevention, Zhengzhou, Henan 450001, P.R. China; ^3^ Department of Physiology, School of Basic Medical Sciences, Zhengzhou University, Zhengzhou, Henan 450001, P.R. China; ^4^ Department of Pathology, Henan Cancer Hospital, Zhengzhou University, Zhengzhou, Henan 450008, P.R. China

**Keywords:** metformin, angiogenesis, ESCC, microenvironment, TECs

## Abstract

Although it has been known that the tumor microenvironment affects angiogenesis, the precise mechanism remains unclear. In this study, we simulated the microenvironment of human esophageal squamous cell carcinoma (ESCC) by tumor conditioned medium (TCM) to assess the influence on normal endothelial cells (NECs). We found that the TCM-induced NECs showed enhanced angiogenic properties, such as migration, invasion and tube formation. Moreover, the TCM-induced NECs expressed tumor endothelial cells (TECs) markers at higher levels, which indicated that TCM probably promoted tumor angiogenesis by coercing NECs to change toward TECs. The microarray gene expression analysis indicated that TCM induced great changes in the genome of NECs and altered many regulatory networks, especially c-MYC and JAK/STAT3 signaling pathway. More importantly, we investigated the anti-angiogenic effect of metformin, and found that metformin abrogated the ESCC microenvironment-induced transition of NECs toward TECs by inhibiting JAK/STAT3/c-MYC signaling pathway. Furthermore, we verified the anti-angiogenic activity of metformin *in vivo* by a human ESCC patient-derived xenograft (PDX) mouse model for the first time. Taken together, our research provides a novel mechanism for the anti-angiogenic effect of metformin, and sets an experimental basis for the development of new anti-angiogenic drugs by blocking the transition of NECs toward TECs, which possibly open new avenues for targeted treatment of cancer.

## INTRODUCTION

Angiogenesis is necessary for tumor growth and metastasis, and constitutes an important point in the control of cancer progression [1]. Notably, the tumor vasculature is composed of a chaotic mixture of abnormal, hierarchically disorganized vessels, which is different from that of normal tissue with respect to organization, structure, and function [2, 3]. Also, some reports have indicated that there were great differences at the molecular and functional levels between tumor endothelial cells (TECs) and normal endothelial cells (NECs), and some TECs specific markers have been found by comparing the gene expression profiles between TECs and their normal counterparts isolated from tissues [4–6]. The abnormal phenotype and function of TECs raises the question of their origin. Compelling evidence indicated that the tumor microenvironment contained a variety of cytokines and microvesicles that governed tumor angiogenesis [7]. However, the underlying mechanism of how the tumor microenvironment promotes the formation of tumor vessels still remains unclear. Hence, in this research, we simulated the microenvironment of ESCC by KYSE450 or KYSE70 culture supernatant and human ESCC tissue homogenate supernatant which were tentatively called tumor conditioned medium (TCM) to investigate the mechanism of ESCC microenvironment-induced angiogenesis.

JAK/STAT3 (Janus kinase/signal transducers and activators of transcription 3) signaling pathway plays essential roles in numerous developmental and homeostatic processes such as cell proliferation, differentiation, apoptosis and angiogenesis [8]. More importantly, it has been known that STAT3 remains constitutively active in approximately 70% of human solid and hematological tumors, and the activated STAT3 promoted the translation of target genes associated with cell cycle, angiogenesis, and invasion/migration [9]. However, whether JAK/STAT3 signaling pathway was activated during the formation of TECs induced by the ESCC microenvironment remained unknown.

Previous epidemiologic studies have documented an association between metformin (a classical drug for type II diabetes) and the reduced cancer incidence and mortality [10]. It is worth noting that the diabetic patients treated with metformin were less likely to develop vascular complications [11]. In addition, accumulated evidences have shown that metformin has the potential to impede tumor angiogenesis [12, 13]. However, the underlying mechanism of how metformin inhibits tumor angiogenesis has not been fully elucidated.

In this study, we found that the ESCC microenvironment promoted tumor angiogenesis by coercing NECs to change toward TECs, which was thought to be one of the origins of TECs. Metformin inhibited the transition of NECs toward TECs by restraining JAK/STAT3/c-MYC signaling pathway. Besides, we verified the anti-angiogenic activity of metformin *in vivo* by a human ESCC PDX tumor-bearing mouse model for the first time.

## RESULTS

### TCM promotes the migration, invasion, tube formation and Dil-Ac-LDL uptake abilities of NECs

**Figure 1:**
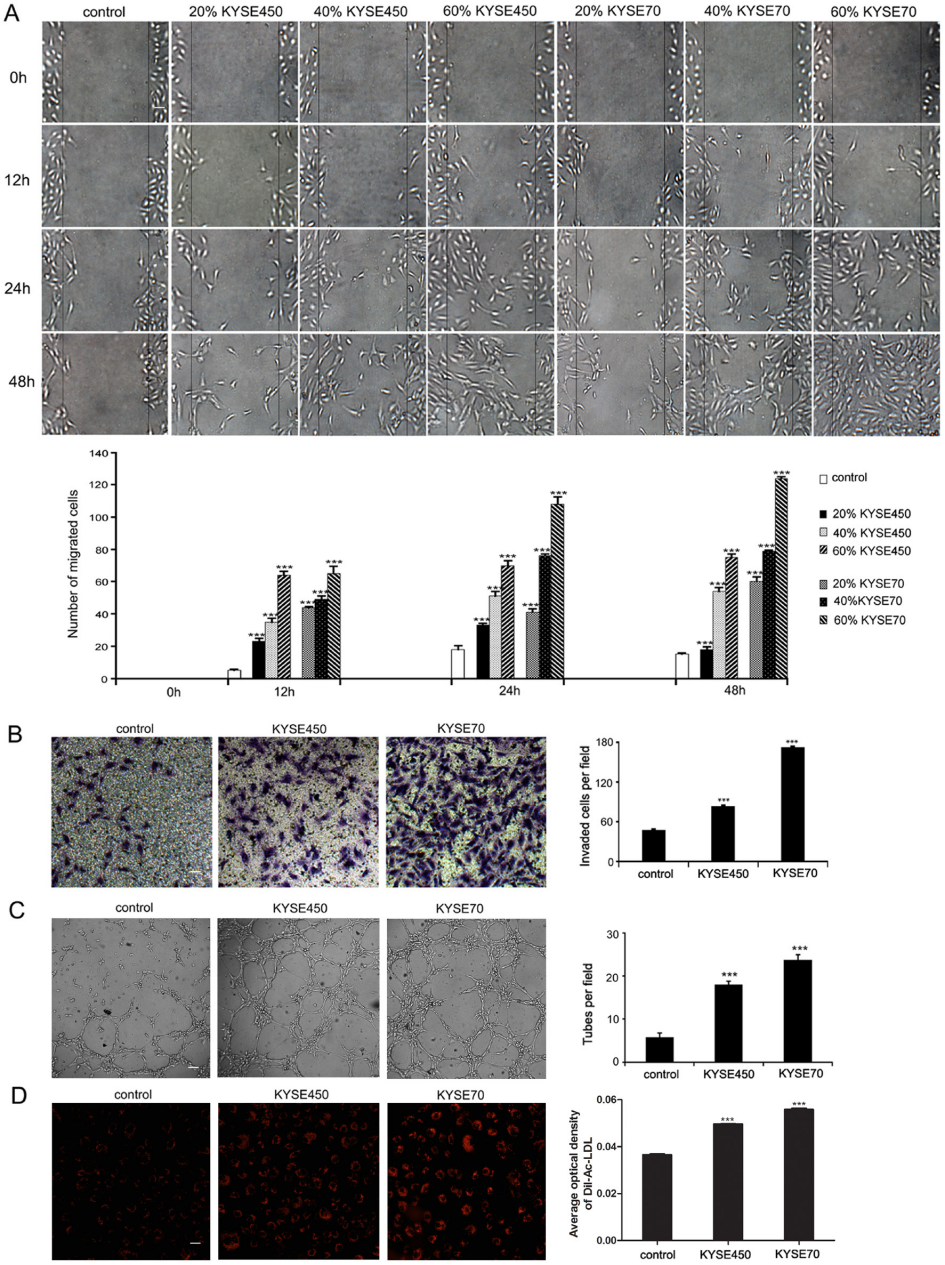
KYSE450 or KYSE70 TCM promoted the migration, invasion, tube formation and Dil-Ac-LDL uptake abilities of NECs **(A)** NECs were plated, scratched and then induced by different concentration of KYSE450 or KYSE70 supernatant as indicated. Photographs were taken at 0, 12, 24 and 48 h after creating the scratch. The number of migrated cells in three random fields was counted (scale bar 40 μm). **(B-D)** NECs were induced by KYSE450 or KYSE70 TCM for 48 h. Then the invasion, tube formation and Dil-Ac-LDL uptake abilities were examined by transwell assay (scale bar 20 μm) (B), tube formation assay (scale bar 40 μm) (C), and Dil-Ac-LDL uptake assay (scale bar 20 μm) (D). Data from three independent experiments are expressed as mean ± SD. *** p < 0.001.

To find proper TCM to simulate the tumor microenvironment, different concentration of KYSE450 or KYSE70 supernatant was used in wound-healing assay. We found that KYSE450 or KYSE70 supernatant promoted the migration ability of NECs in a time and concentration dependent manner (Figure 1A). Therefore, TCM comprised 60% KYSE450 or KYSE70 supernatant and 40% FBS free endothelial cell medium was chosen to do the following research. As migration and invasion are two key steps for endothelial cells (ECs) to form new blood vessels during angiogenesis processes [14], we performed transwell assay to determine the effect of TCM on the invasion ability of NECs. Compared with the control group, the invasion ability of NECs in the KYSE450 or KYSE70 TCM-induced group was significantly enhanced. Moreover, NECs in the KSYE70 TCM-induced group had stronger invasion ability than that in the KYSE450 TCM-induced group (Figure 1B). ECs move to new locations by migration and invasion, and finally evolve into vascular networks by forming tubes. As the results shown in tube formation assay, NECs in the KYSE450 or KYSE70 TCM-induced group developed more tubes than NECs in the control group (Figure 1C). Interestingly, the Dil-Ac-LDL uptake ability of NECs was also enhanced after induction by KYSE450 or KYSE70 TCM (Figure 1D). Human ESCC tissue homogenate TCM was further utilized to simulate the tumor microenvironment. The abilities of migration, invasion, tube formation and Dil-Ac-LDL uptake of NECs were enhanced after induction by TCM from ESCC tissue homogenate compared with that from peri-carcinoma tissue homogenate (Supplementary Figure 1). Taken together, these data reveal that the ESCC microenvironment enhances the angiogenic properties of NECs.

### TCM promotes the transition of NECs toward TECs

**Figure 2:**
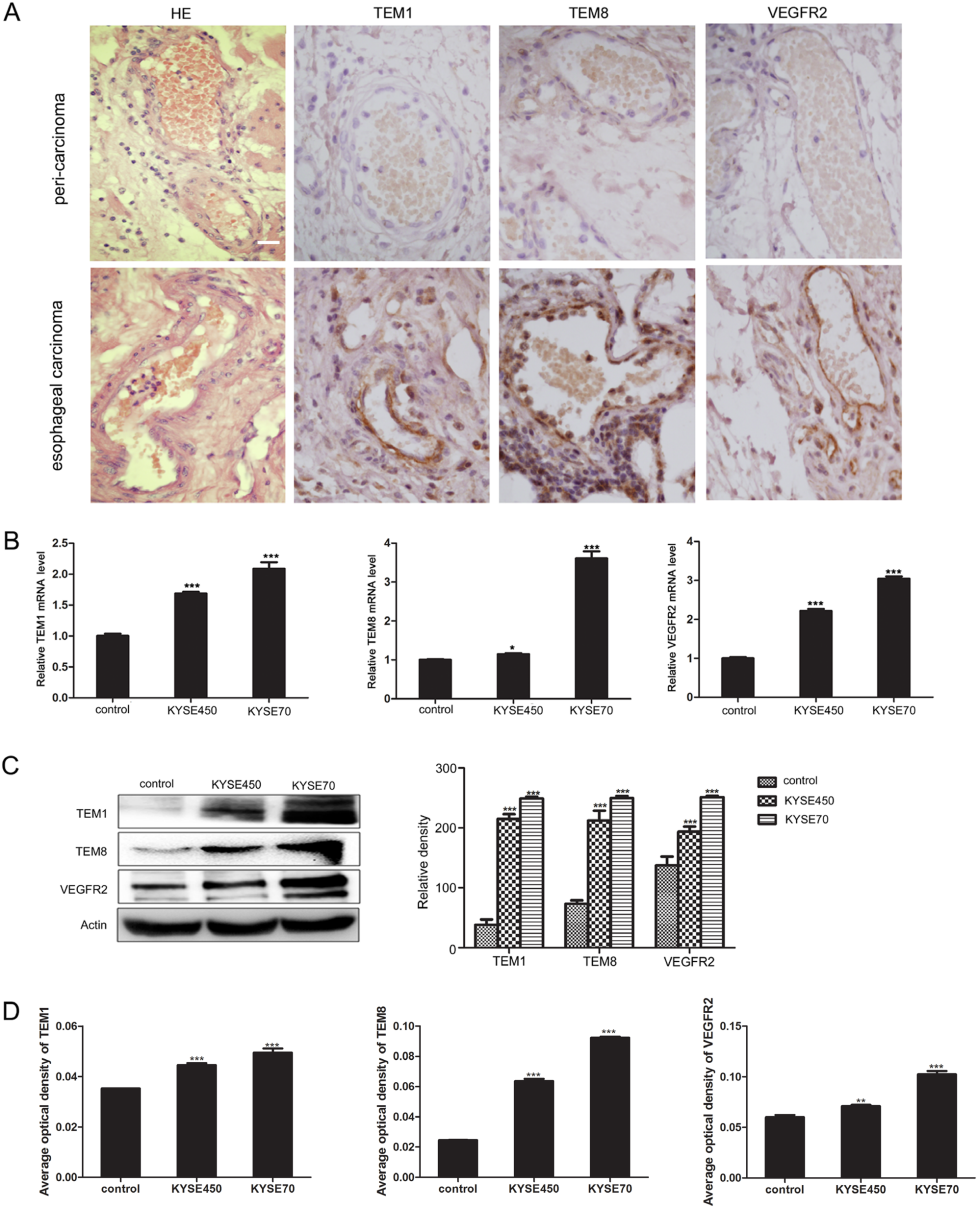
KYSE450 or KYSE70 TCM induced NECs to have the characteristics of TECs **(A)** Immunohistochemistry validated the TECs markers (TEM1, TEM8 and VEGFR2) in esophageal carcinoma and peri-carcinoma tissue. TEM1, TEM8 and VEGFR2 were preferentially expressed in vascular endothelial cells of esophageal carcinoma tissue (scale bar 20 μm). **(B-D)** NECs were induced by KYSE450 or KYSE70 TCM for 48 h. The relative mRNA levels of TECs markers were examined by qRT-PCR (B). The protein levels of TECs markers were detected by Western blot (C) and immunofluoresence (D). Results are expressed as mean ± SD. * p < 0.05, ** p < 0.01, *** p < 0.001.

Firstly, we detected the expression of TEM1, TEM8 and VEGFR2 using immunohistochemistry on paraffin section of human esophageal carcinoma tissue and peri-carcinoma tissue. The results showed that TEM1, TEM8 and VEGFR2 were expressed specifically in vascular ECs of tumor tissue (Figure 2A), which were in accordance with previous reports and proved that they were TECs markers [4, 15]. Then we explored the influence of KYSE450 or KYSE70 TCM on the molecular expression of NECs. In comparison with control, NECs in the KYSE450 or KYSE70 TCM-induced group highly expressed TECs markers both at mRNA and protein levels. Furthermore, the mRNA and protein levels of TECs markers in KYSE70 TCM-induced NECs were enhanced more remarkable than that in KYSE450 TCM-induced NECs (Figure 2B-2D). For NECs induced by the ESCC tissue homogenate TCM, the results were in accordance with that induced by KYSE450 or KYSE70 TCM (Supplementary Figure 2). These data suggest that the ESCC microenvironment promotes the transition of NECs toward TECs.

### Increased expression of c-MYC is a key factor that promotes the transition of NECs toward TECs induced by TCM

**Figure 3:**
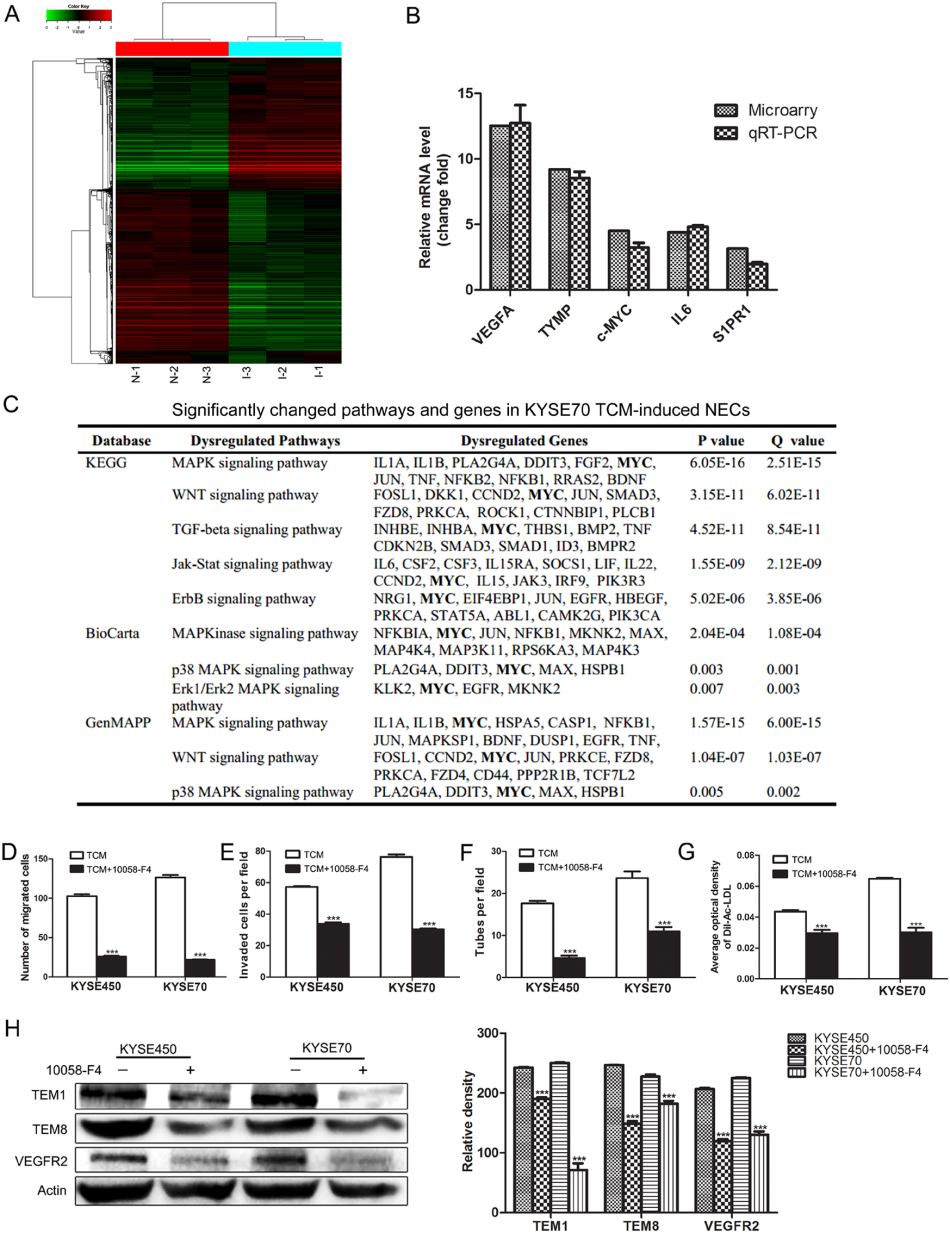
c-MYC played a key role in promoting the transition of NECs toward TECs induced by KYSE450 or KYSE70 TCM **(A)** Microarray analysis was performed on total RNA isolated from NECs (N-1, N-2, N-3) and KYSE70 TCM-induced NECs (I-1, I-2, I-3). Hierarchical clustering assays showed DEGs in KYSE70 TCM-induced NECs compared with NECs. 0 indicated by pure black represents no change from the median gene expression level in all samples. -3 indicated by pure green represents relatively lower expression. +3.0 indicated by pure red represents relatively higher expression. **(B)** qRT-PCR validation of the up-regulated genes in KYSE70 TCM-induced NECs. **(C)** Molecule annotation system analyzed the significantly changed pathways and genes in KYSE70 TCM-induced NECs. **(D-G)** 10058-F4 inhibited the transition of NECs toward TECs induced by KYSE450 or KYSE70 TCM. 10058-F4 inhibited the migration (D), invasion (E), tube formation (F) and Dil-Ac-LDL uptake (G) abilities of KYSE450 or KYSE70 TCM-induced NECs. **(H)** Analysis of the protein levels of TECs markers (TEM1, TEM8, VEGFR2) in NECs induced by KYSE450 or KYSE70 TCM with or without 10058-F4 by Western blot. Data are shown as mean ± SD. *** p < 0.001.

To further investigate the mechanism of tumor angiogenesis promoted by TCM, we performed microarray analysis on NECs (N-1, N-2, N-3) and KYSE70 TCM-induced NECs (I-1, I-2, I-3). We identified a subset of 3769 differential expressed genes (DEGs) in KYSE70 TCM-induced NECs compared with control NECs, including 1609 up-regulated genes and 2160 down-regulated genes. Hierarchical clustering assay showed all the detected genes (Figure 3A). qRT-PCR was performed to validate the microarray results by detecting the relative mRNA change fold of 5 selected DEGs (VEGFA, TYMP, c-MYC, IL6 and S1PR1). The results of qRT-PCR were in accordance with the results of microarray assay (Figure 3B). The bioinformatics analysis on these DEGs showed that the significantly changed pathways mainly associated with angiogenesis and cell differentiation. Notably, MYC was involved in many significantly changed pathways according to database analysis of KYGG, BioCarta and GenMAPP (Figure 3C). Previous researches have demonstrated that c-MYC was essential for vasculogenesis and angiogenesis during development and tumor progression [16, 17]. Thus, we speculated that the enhanced expression of c-MYC might play an essential role during the transition of NECs toward TECs induced by TCM. To elucidate the crucial role of c-MYC, 10058-F4, a small-molecule inhibitor of c-MYC was used. The results showed that 10058-F4 markedly attenuated the enhanced abilities of migration, invasion, tube formation and Dil-Ac-LDL uptake of TCM-induced NECs (Figure 3D-3G). In addition, the TCM-induced overexpression of TECs markers in NECs were significantly reduced by 10058-F4 (Figure 3H and Supplementary Figure 3). These data indicate that the increased expression of c-MYC is a key factor that promotes the transition of NECs toward TECs induced by the ESCC microenvironment.

### JAK/STAT3 signaling pathway is activated during the TCM-mediated transition of NECs toward TECs

**Figure 4:**
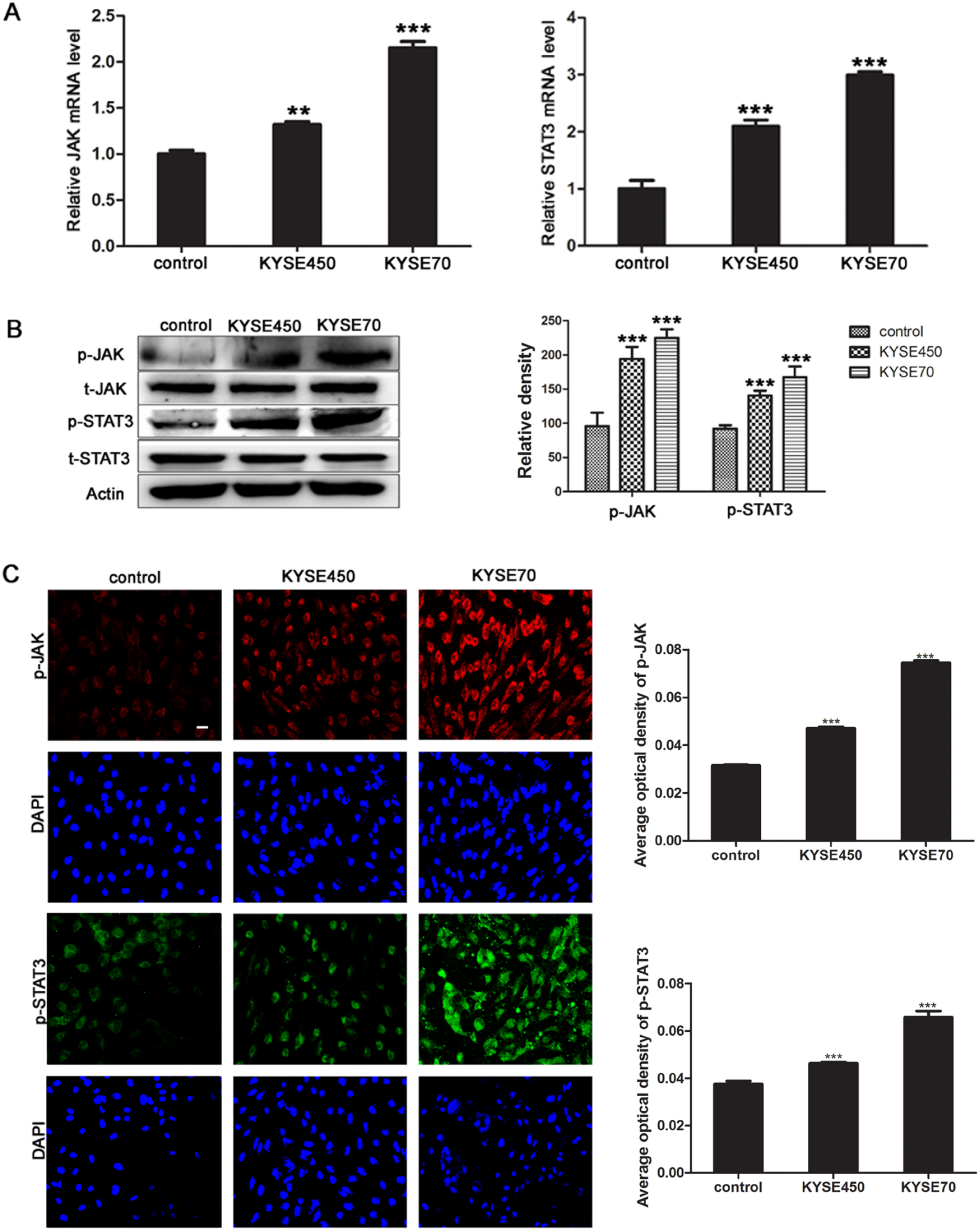
JAK/STAT3 signaling pathway was activated during the transition of NECs toward TECs induced by KYSE450 or KYSE70 TCM **(A)** NECs were induced by KYSE450 or KYSE70 TCM for 48 h. Relative mRNA levels of JAK and STAT3 were analyzed by qRT-PCR. **(B** and **C)** The protein levels of p-JAK and p-STAT3 were detected by Western blot (B) and immunofluoresence (scale bar 50 μm) (C). Results are expressed as mean ± SD. ** p < 0.01, *** p < 0.001.

According to the results of microarray assay, the pathway analysis showed that many pathways were significantly changed, especially JAK/STAT3 pathway. Together with that c-MYC was one of STAT3 target genes, and was essential for tumor angiogenesis, we wondered whether JAK/STAT3 signaling pathway was aberrant activated during the transition of NECs toward TECs. The results showed that the relative mRNA levels of JAK and STAT3 in KYSE450 or KYSE70 TCM-induced NECs were significantly increased (Figure 4A). Meanwhile, the phosphorylated JAK and STAT3 proteins were also expressed at higher levels in KYSE450 or KYSE70 TCM-induced NECs compared with control NECs (Figure 4B-4C). Then, the ESCC tissue homogenate TCM was further used to induce NECs, the results were consistent with that induced by KYSE450 or KYSE70 TCM (Supplementary Figure 4). Taken together, the results demonstrate that JAK/STAT3 signaling pathway is activated during the transition of NECs toward TECs induced by the ESCC microenvironment.

### Metformin inhibits the TCM-induced transition of NECs toward TECs by blocking JAK/STAT3/c-MYC pathway

**Figure 5:**
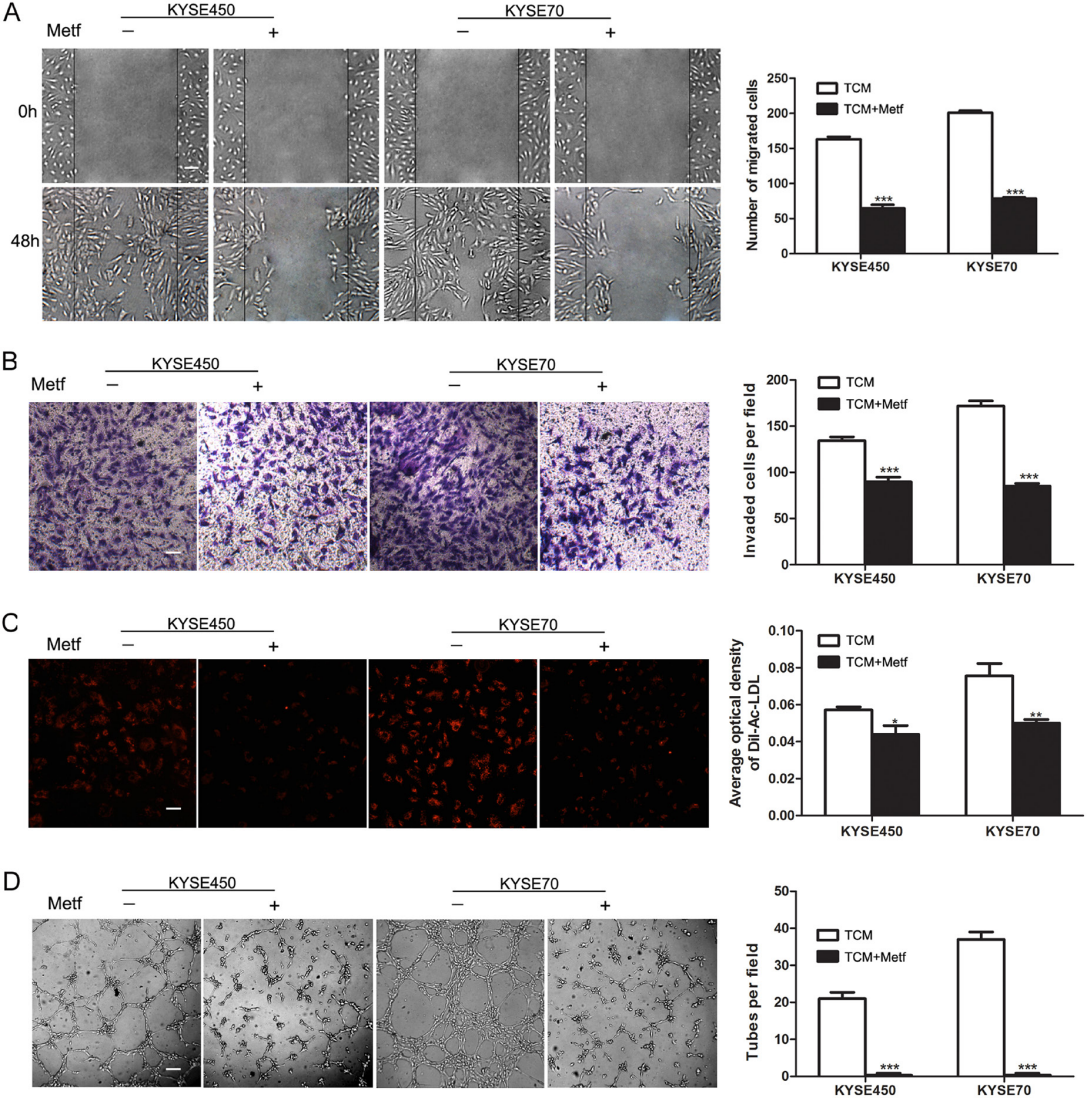
Metformin inhibited the migration, invasion, tube formation and Dil-Ac-LDL uptake abilities of KYSE450 or KYSE70 TCM-induced NECs **(A)** NECs were plated, scratched and induced by KYSE450 or KYSE70 TCM with or without metformin for 48 h (10 mM). Photographs were taken at 0 h and 48 h (scale bar 40 μm). **(B-D)** NECs were induced by KYSE450 or KYSE70 TCM with or without metformin for 48 h. The effect of metformin on the invasion, Dil-Ac-LDL uptake and tube formation abilities of KYSE450 or KYSE70 TCM-induced NECs were measured by transwell assay (scale bar 20 μm) (B), Dil-Ac-LDL uptake assay (scale bar 20 μm) (C), and tube formation assay (scale bar 40 μm). Data are shown as mean ± SD. * p < 0.05, ** p < 0.01, *** p < 0.001.

Although many studies have indicated the anti-angiogenic effect of metformin, the mechanism was still unclear [12]. Given the above data, we wondered whether metformin suppressed tumor angiogenesis by blocking the transition of NECs toward TECs induced by TCM. The characteristics of NECs were detected after induction by KYSE450 or KYSE70 TCM with or without metformin for 48 h. The results showed that the enhanced abilities of migration, invasion, Dil-Ac-LDL uptake and tube formation of KYSE450 or KYSE70 TCM-induced NECs were significantly abrogated by metformin (Figure 5A-5D).

**Figure 6:**
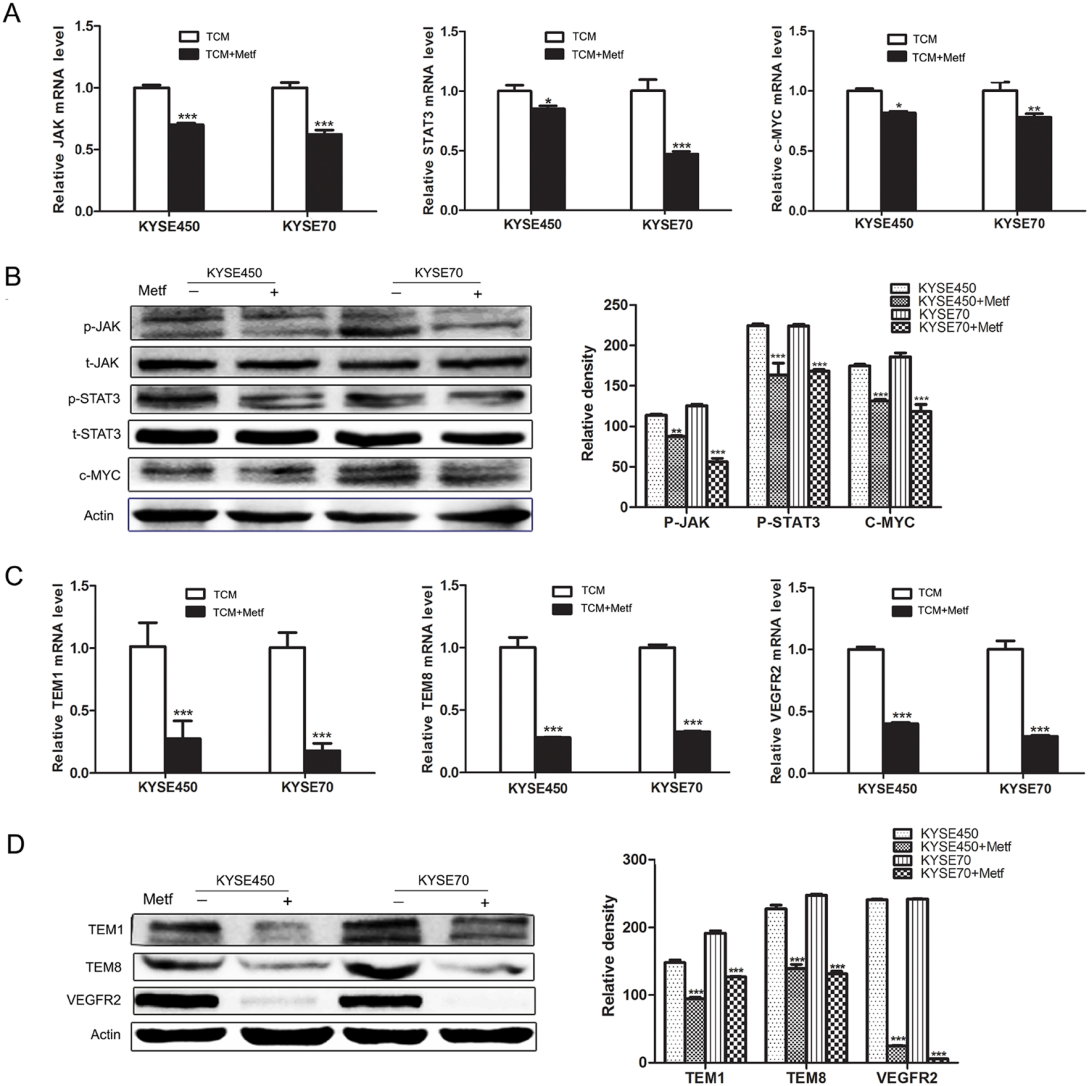
Metformin inhibited the transition of NECs toward TECs by blocking JAK/STAT3/c-MYC signaling pathway **(A)** NECs were induced by KYSE450 or KYSE70 TCM with or without metformin for 48 h. The relative mRNA levels of JAK, STAT3 and c-MYC were analyzed by qRT-PCR. **(B)** Western blot was done to detect the expression levels of indicated proteins. **(C** and **D)** The mRNA and protein levels of TECs markers (TEM1, TEM8 and VEGFR2) were detected by qRT-PCR (C) and Western blot (D). Data are shown as mean ± SD. * p < 0.05, ** p < 0.01, *** p < 0.001.

The above data have proved that JAK/STAT3/c-MYC signaling pathway was activated during the transition of NECs toward TECs. To further explore the mechanism of the anti-angiogenic activity of metformin, we detected its influence on JAK/STAT3/c-MYC signaling pathway. The results showed that metformin decreased the relative mRNA levels of JAK, STAT3 and c-MYC in TCM-induced NECs (Figure 6A). More importantly, metformin reduced the phosphorylation of JAK and STAT3, and decreased the protein level of c-MYC (Figure 6B and Supplementary Figure 5). Then we assessed the effects of metformin on the transition of NECs toward TECs. The results showed that TCM-induced NECs in the metformin-treated group had lower expressions of TECs markers (TEM1, TEM8 and VEGFR2) compared with that without metformin treatment (Figure 6C-6D and Supplementary Figure 6). These data indicate that metformin abrogates the TCM-induced transition of NECs toward TECs by blocking JAK/STAT3/c-MYC signaling pathway, and c-MYC may be an important molecular target of metformin.

### Metformin inhibits tumor angiogenesis in the human ESCC PDX mouse model

**Figure 7:**
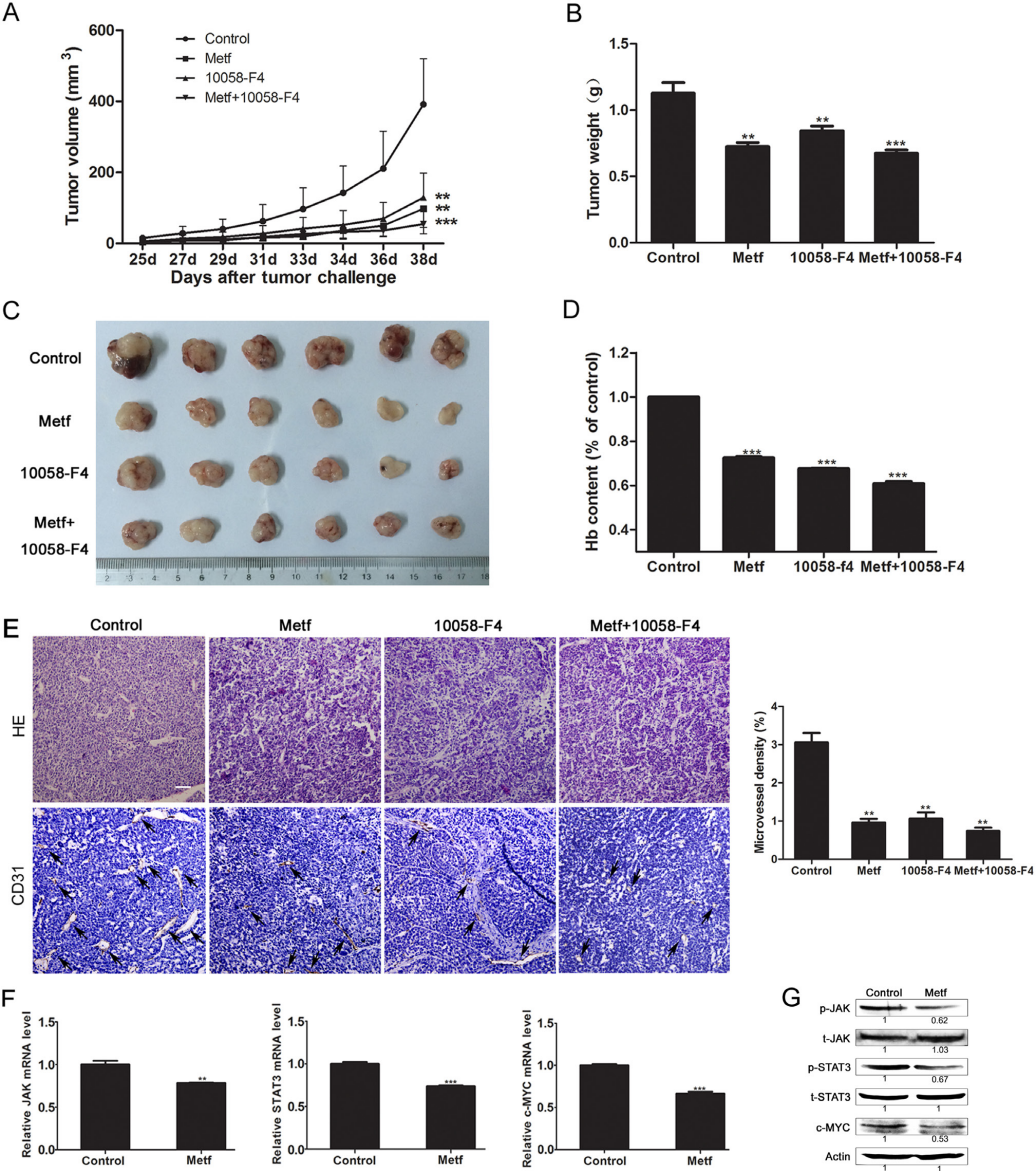
Metformin showed anti-tumor effect by suppressing tumor angiogenesis in the ESCC PDX mouse model **(A)** Tumor bearing mice were given metformin (Metf), 10058-F4 or metformin combination with 10058-F4 (Metf+10058-F4), then the tumor volume was measured every other day. **(B-D)** The tumors of various groups were resected after 14 days of treatment, the weight (B), size (C), and hemoglobin level (D) were measured. **(E)** Immunohistochemical analysis of vascularization by staining tumor tissue sections with anti-CD31 antibody and quantification of microvessel density (scale bar 20 μm). **(F)** The relative mRNA levels of JAK, STAT3 and c-MYC in tumor tissues of the metformin group and control group were determined by qRT-PCR. **(G)** The expression levels of indicated proteins in tumor tissues were detected by Western blot. The relative expression level of each protein in the metformin group was normalized to the control group. Data are shown as mean ± SD. ** p < 0.01, *** p < 0.001.

To investigate the effect of metformin on suppressing tumor angiogenesis *in vivo*, a human ESCC PDX mouse model was used. The results showed that metformin profoundly reduced the tumor size. Importantly, metformin had a similar anti-tumor effect with 10058-F4, and a lower toxicity than 10058-F4. Interestingly, the anti-tumor effect was not enhanced obviously when treating mice with metformin combination with 10058-F4 (Figure 7A-7C). Then we evaluated the status of angiogenesis in tumor specimens by determining the hemoglobin content and microvessel density. The results showed that metformin and 10058-F4 exhibited comparable inhibitory effect on tumor angiogenesis in mice. While the inhibitory effect was not enhanced obviously in mice treated with metformin combination with 10058-F4 compared with that treated with either single agent (Figure 7D-7E). To verify our hypothesis that metformin inhibits tumor angiogenesis by suppressing JAK/STAT3/c-MYC signaling pathway, we detected the expression levels of related genes and proteins in tumor specimens from mice. As expected, metformin significantly decreased the relative mRNA levels of JAK, STAT3 and c-MYC in tumor tissues (Figure 7F). Furthermore, metformin inhibited the c-MYC protein expression level, and reduced the phosphorylation levels of JAK and STAT3 (Figure 7G). Overall, these results suggest that metformin inhibits angiogenesis of ESCC *in vivo* by blocking JAK/STAT3/c-MYC signaling pathway.

## DISCUSSION

Tumor microenvironment is the product of a crosstalk between different cells types, which plays a critical role in promoting the initiation and progression of malignancy [18]. Several reviews have been published shown that the tumor microenvironment and factors therein influenced angiogenesis, while the mechanism still remains to be illustrated [19–21]. In this research, we simulated the ESCC microenvironment by TCM to induce NECs, and found that TCM promoted the angiogenic properties of NECs, such as migration, invasion, tube formation and Dil-Ac-LDL uptake. It has been reported that TECs were distinct from normal counterparts both at the molecular and functional levels [15, 22]. Our following research indicated that the TCM-induced NECs expressed TECs markers at higher levels. Thus, it is reasonable to assume that the ESCC microenvironment promotes tumor angiogenesis by coercing NECs to change toward TECs. Since it is difficult to isolate TECs from tumor tissue, most studies on tumor angiogenesis were carried out by using NECs, such as HUVECs and human dermal microvascular endothelial cells (HDMECs) [23, 24]. Our finding that NECs induced by TCM have the properties of TECs provides a new and convenient method to conduct further research on tumor angiogenesis, especially drugs target to TECs.

Another intriguing result of our study was the global effect of TCM on the transcriptome of NECs, with the modulation of 3769 genes. Many genes account for several aspects of the endothelial response, such as VEGFA, IL6 and TYMP for tumor angiogenesis, and S1PR1 for development and cell differentiation [7, 25]. Our data also indicated that TCM played great influence on the whole gene network of NECs, resulting in the modulation of signaling pathways that mediated the ECs response. It is noteworthy that c-MYC was involved in many significantly changed signaling cascades in NECs induced by TCM. It has been well known that the oncoprotein c-MYC was overexpressed in a wide range of human cancers [26]. Previous studies have demonstrated that c-MYC regulated other proteins involved in angiogenesis, which provided evidence to show c-MYC as a master regulator of vascular remodeling [17, 27]. In our study, c-MYC was highly expressed in TCM-induced NECs, it is reasonable to speculate that c-MYC might act as a key mediator during the transition of NECs toward TECs. This speculation was then supported by the results of our study that the inhibitor of c-MYC, 10058-F4, antagonized the phenotypic and functional changes of NECs induced by TCM.

Among the activated signaling cascades that c-MYC involved, JAK/STAT3 signaling pathway attracted more attention for its crucial role in ECs activation and pathological angiogenesis [28]. Moreover, STAT3 was aberrant activated in ESCC, and was associated with poor prognosis of patients [29, 30]. Although the JAK/STAT3 signaling pathway is well documented in cancer progression, there are few reports on its physiological significance in the formation of TECs. Here, our results showed that JAK/STAT3 signaling pathway was activated during the ESCC microenvironment-induced transition of NECs toward TECs. In this context, blocking JAK/STAT3 signaling pathway would be an effective anti-angiogenic therapeutic method for ESCC.

Previous study has demonstrated that metformin inhibited ESCC growth by downregulating STAT3 signaling pathway [31]. In addition, metformin showed anti-angiogenic activity by modulating different mediators that governing the angiogenic process [13, 32]. However, the precise mechanism for the anti-angiogenic property of metformin still needs to be further defined. In the present study, we found that metformin significantly suppressed the migration, invasion, Dil-Ac-LDL uptake and tube formation abilities of TCM-induced NECs. Mechanistically, metformin inhibited the TCM-induced transition of NECs toward TECs through inhibiting JAK/STAT3/c-MYC signaling pathway. These data provide a novel explanation for the anti-angiogenic activity of metformin.

Then we used a human ESCC PDX mouse model to investigate the anti-angiogenic effect of metformin *in vivo*. It has been known that the tumors of PDX model maintained the original molecular characteristic and heterogeneity of the original patient tumors [33]. Moreover, the PDX mouse model was considered to be highly relevant to real human tumor growth as it developed a functional stroma and microenvironment [34]. Hence, the results we got from the PDX model are more convincing than tumor cell line xenograft model, and have stronger predictive power of clinical transformation. In this study, we found that metformin exerted similar effect with 10058-F4 on inhibiting tumor growth and tumor angiogenesis in the ESCC PDX mouse model. Although it has been reported that 10058-F4 acted as a tumor suppressor in several different tumors, there is still a long way for its clinical application [35]. Different from 10058-F4, metformin has been used in clinic for years, and the security has been widely approved. Therefore, our research sets the experimental basis for the clinical application of metformin as an anti-angiogenic drug.

In summary, we conclude that the ESCC microenvironment promotes tumor angiogenesis by coercing NECs to change toward TECs, with the activation of JAK/STAT3/c-MYC signaling pathway. Metformin exerts anti-angiogenic effect by suppressing this process, which is a novel mechanism for metformin in anti-angiogenesis. Our findings provide a fresh perspective on tumor angiogenesis, and set the stage for exploring clinically relevant drugs that suppressing tumor angiogenesis through blocking the transition of NECs toward TECs, which possibly open new avenues for targeted treatment of cancer.

## MATERIALS AND METHODS

### Cell culture

KYSE450 and KYSE70 are human esophageal squamous cancer cell lines in high and poor differentiated, respectively. KYSE450 and KYSE70 were cultured in RPMI-1640 (Biological Industries, Kibbutz Beit Haemek, Israel) medium with 10% FBS. Human umbilical vein endothelial cells (HUVECs) were cultured in endothelial cell medium (ScienCell, Carlsbad, CA, USA). All cell lines were cultured at 37°C under 5% CO_2_.

### Preparation of tumor conditioned medium (TCM)

KYSE450 or KYSE70 were cultured in 10 cm dishes and replenished with 5 ml fresh medium after reaching 60-80% confluence. Then the supernatant was collected and centrifuged after 24 h incubation and stored at -20°C. The ESCC cell line TCM was composed by 60% KYSE450 or KYSE70 supernatant and 40% FBS free endothelial cell medium. In the experimental group, NECs were induced by ESCC cell line TCM for 48 h. While in the control group, NECs were induced only by FBS free endothelial cell medium at the same condition.

To further simulate the ESCC microenvironment, we used the human ESCC tissue homogenate supernatant. The tissue homogenate supernatant of corresponding peri-carcinoma was used as control. The preparation of tissue homogenate supernatant of ESCC and peri-carcinoma was according to previous report [36]. Briefly, tumor specimen of human ESCC and peri-carcinoma tissues (> 5 cm) obtained intraoperatively from patients were weighed, cut into pieces and ground by glass pestle. After that, the homogenate was added FBS free RPMI-1640 medium at 0.2 g/ml, then centrifuged at 13,000 rpm for 30 min. Finally, the ESCC or peri-carcinoma tissue homogenate supernatant was collected, and stored at -20°C. The ESCC tissue homogenate TCM was composed by 40% supernatant and 60% FBS free endothelial cell medium. Patients whose tumor samples were involved in this study were completely informed and provided consent.

### Wound-healing assay

NECs were cultured in 12-well plates and incubated overnight to a density of 60% -70%. Then cell monolayer was carefully scratched with a 200-μl pipette tip to draw a straight “wound” in each well and washed twice with PBS. Different concentration (20%, 40% or 60%) of TCM was applied to culture cells for 48 h at 37°C with 5% CO_2_. The wounded areas were imaged using a microscope (Olympus, Tokyo, Japan) at the indicated time point (0 h, 12 h, 24 h and 48 h). The number of migrated cells in per field was counted.

### Transwell assay

The membrane surface of transwell (pore size: 8 μm, Corning, NY, USA) was precoated with diluted matrigel (1:4, BD Biosciences, San Jose, CA, USA) and incubated at 37°C for 2 h. NECs (3.75×10^4^ cells in FBS free endothelial cell medium) were seeded onto the upper chamber, and the bottom chamber was filled with completed endothelial cell medium. Then the chambers were incubated at 37°C for 24 h. The invaded cells were stained with crystal violet after being fixed with 10% TCA for 1 h, then imaged by an inverted microscope (Olympus, Tokyo, Japan).

### Tube formation assay

Chilled liquid matrigel was dispensed onto 96-well plates (50 μl per well) and allowed to solidify at 37°C for 1 h. Then NECs (2×10^4^ cells per well) were seeded onto the gel and cultured at 37°C with 5% CO_2_ for 4 h. The enclosed networks of complete tubes from three randomly chosen fields were counted and photographed using a microscope (Olympus, Tokyo, Japan).

### Dil-labeled acetylated low-density lipoprotein (Dil-Ac-LDL) uptake assay

NECs (3,000 per well) were seeded in 96-well plates for adherence overnight, and induced by TCM for 48 h. Then the Dil-Ac-LDL (10 μg/ml; Biomedical Technologies, Stoughton, MA, USA) was added to each well, and the cells were incubated at 37°C for another 4 h. The medium containing Dil-Ac-LDL was removed and the cells were washed three times with PBS. Then the cells were observed and photographed using a fluorescence microscope (Olympus, Tokyo, Japan). The average optical density of Dil-Ac-LDL was calculated with the formula: the value of Integrated density / the value of Area. The values of Integrated density and Area were measured by image J software.

### Quantitative real-time PCR (qRT-PCR)

Total RNA of NECs was extracted using the TRIzol (Invitrogen, Carlsbad, CA, USA). One microgram of total RNA was reverse-transcribed using a RT reagent kit (TaKaRa, Tokyo, Japan). The cDNA was amplified with a 7500 Fast Real-time PCR System (Applied Biosystems, NY, USA). The data analysis was performed on 7500 software v2.0.5. The comparative threshold cycle (Ct) method, i.e., 2^-ΔΔCt^ was used to calculate fold amplification.

### Western blot

Protein extracts for Western blot were prepared with lysis buffer (1mM PMSF, 1mM NaF, 1mM Na_3_VO_4_, protease inhibitor). The protein concentration was determined using a BCA protein assay kit (Beyotime, Shanghai, China). Western blot was performed as previously described [37]. Antibodies against STAT3, p-STAT3, JAK2, p-JAK2, TEM1, VEGFR2 and β-Actin were purchased from Santa Cruz Biotechnology (Santa Cruz, Dallas, TX, USA). Antibodies against c-MYC and TEM8 were purchased from Abcam (Abcam, Cambridge, UK). Protein bands were visualized using a chemiluminescence detection kit (Beyotime, Shanghai, China).

### Immunofluorescence

Immunofluorescence was performed as previously described [38]. Briefly, cells were fixed with 4% paraformaldehyde for 30 min at room temperature. After being blocked with 1% BSA-PBST (Solarbio, Beijing, China) for 1.5 h, the cells were incubated with primary antibody (1:50) overnight at 4°C. The primary antibodies used in immunofluorescence were as same as that used in Western blot. The 594-conjugated goat anti-rabbit IgG or 488-conjugated goat anti-mouse IgG (Origene, Beijing, China) was used as the secondary antibody and incubated cells for 1.5 h at room temperature. All images were captured by a laser scanning confocal microscope (Olympus, Tokyo, Japan). The average optical density of individual protein was calculated with the formula: the value of Integrated density / the value of Area. The values of Integrated density and Area were measured by image J software.

### Microarray gene expression analysis

The experimental design and microarray data analysis were conducted according to previous reports [25, 39]. NECs and KYSE70 TCM induced-NECs RNA extraction and microarray hybridization were performed at CapitalBio Technology Company (Beijing, China) according to the standard procedure. Overall, six microarray chips were analyzed in this study. The obtained scanned images were analyzed using Affymetrix GeneChip Operating Software (GCOS 1.4). To compare the differential expressed genes (DEGs), we applied the Significance Analysis of Microarrays Software (SAM version 3.02, Stanford University, Stanford, CA, USA). The algorithm used to sort the statistically significant DEGs was a modified t-test, and the criteria for DEGs were q-value < 0.05 and fold change > 2.0 or < 0.5. DEGs were further subjected to the CapitalBio^®^ Molecule Annotation System V3.0 (MAS3.0) for gene ontology (GO) and KEGG pathway analysis. As for GO and KEGG pathways, the p-value and q-value were calculated to select significantly affected pathways.

### *In vivo* PDX study

The ESCC PDX model was established as previous report [33]. Briefly, fresh human ESCC fragments obtained intraoperatively from patients were subcutaneously implanted into 6 to 8 week-old female CB17/SCID mice. When the xenograft ESCC tumor reached about 1,500 mm^3^, the mouse was sacrificed, and the tumor was divided into 0.1-0.2 g fragments and implanted into additional mice for further generations. After three consecutive mouse-to-mouse passages, we got enough tumor bearing mice to do the following research. Once the tumor volume reached approximately 25 mm^3^, mice were randomly divided into 4 groups (n = 10 mice per group): Control group, Metformin (200 mg/kg) group, 10058-F4 (15 mg/kg) group and Metformin combination with 10058-F4 group. Body weight and tumor measurement were performed every other day. Tumor volume was calculated using the formula V=0.5ab^2^, with “a” as the long diameter in millimeters and “b” as the short diameter in millimeters. This study was approved by Ethics Committee of Zhengzhou University and patients whose tumor samples were involved in this study were completely informed and provided consent.

### Hemoglobin assay

Tumor tissue was weighed and homogenized in 1 ml Drabkin reagent (Sigma-Aldrich, St. Louis, MO, USA) and centrifuged at 12,000×g for 20 min. The supernatant was filtered through a 0.22 μm millpore filter. The hemoglobin concentration of samples was determined spectrophotometrically by measuring absorbance at 540 nm using an ELISA plate reader (Thermo Fisher Scientific, Waltham, MA, USA). The relative content of hemoglobin in the tumor tissue was compared with the control group.

### Immunohistochemistry

Tumor tissues were fixed in 10% formalin after being resected, embedded in paraffin and cut into 4 μm sections. The following procedure was performed as previously described [40]. Slides were incubated with primary antibodies against TEM1, TEM8, VEGFR2 or CD31 (Abcam, Cambridge, UK) at 4°C overnight. Then the slides were incubated with HRP-IgG secondary antibody at 37°C for 15 min, followed by developing with diaminobenzidine and counterstained with haematoxylin. Hematoxylin-eosin (H&E) staining was conducted according to the standard histological procedure.

### Statistical analysis

All analyses were performed using GraphPad PRISM software version 5.0 (GraphPad Software, USA). Statistical analyses and significance were analyzed by one-way ANOVA with Tukey’s post-hoc test. In all comparisons, p < 0.05 was considered statistically significant.

## SUPPLEMENTARY MATERIALS FIGURES


