# Supplementary material for: Metformin inhibits esophageal squamous cell carcinoma-induced angiogenesis by suppressing JAK/STAT3 signaling pathway
Source: Oncotarget. 2017 Aug 18;8(43):74673–87. doi: 10.18632/oncotarget.20341 (PMC5650371; doi:10.18632/oncotarget.20341)
Supplement: Supplementary file 1 [file oncotarget-08-74673-s001.pdf]

Metformin inhibits esophageal squamous cell carcinoma-induced angiogenesis by suppressing JAK/STAT3 signaling pathway

SUPPLEMENTARY MATERIALS

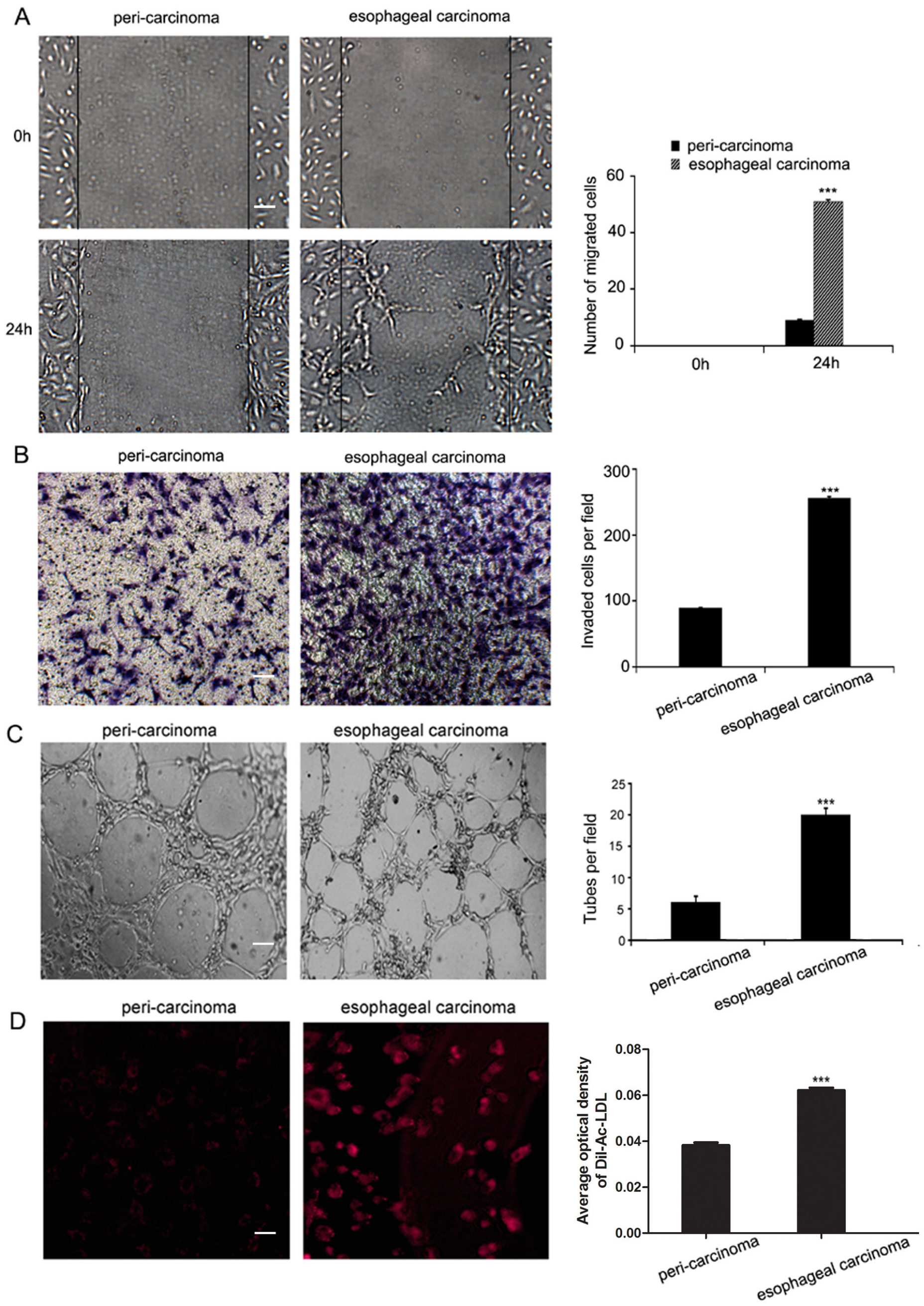

Supplementary Figure 1: ESCC tissue homogenate TCM promoted the migration, invasion, tube formation and Dil-Ac-LDL uptake abilities of NECs. (A-D) NECs were cultured in esophageal carcinoma or peri-carcinoma tissue homogenate TCM for 48 h, then the migration, invasion, tube formation and Dil-Ac-LDL uptake abilities were examined by wound-healing assay (scale bar 40μm) (A), transwell assay (scale bar 20μm) (B), tube formation assay (scale bar 40μm) (C), and Dil-Ac-LDL uptake assay (scale bar 20μm) (D). Data from three independent experiments are expressed as mean±SD. *** $p < 0.001$.

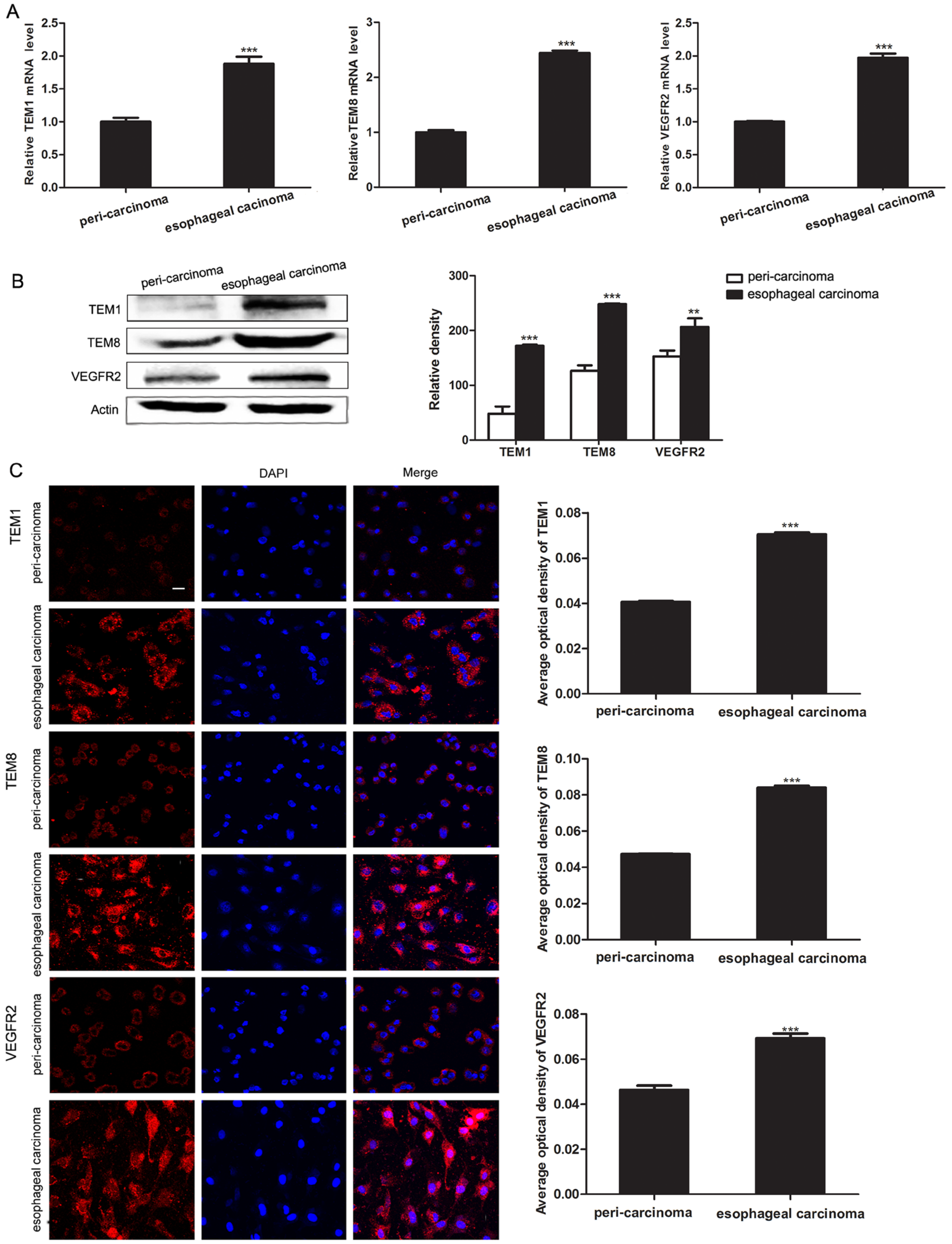

Supplementary Figure 2: ESCC tissue homogenate TCM promoted NECs to have the characteristic of TECs. (A-C) NECs were induced by esophageal carcinoma or peri-carcinoma tissue homogenate TCM for 48 h. The expression levels of TECs markers (TEM1, TEM8 and VEGFR2) were detected by qRT-PCR (A), Western blot (B) and immunofluoresence (scale bar 50μm) (C). Results are expressed as mean±SD. ** $p < 0.01$, *** $p < 0.001$.

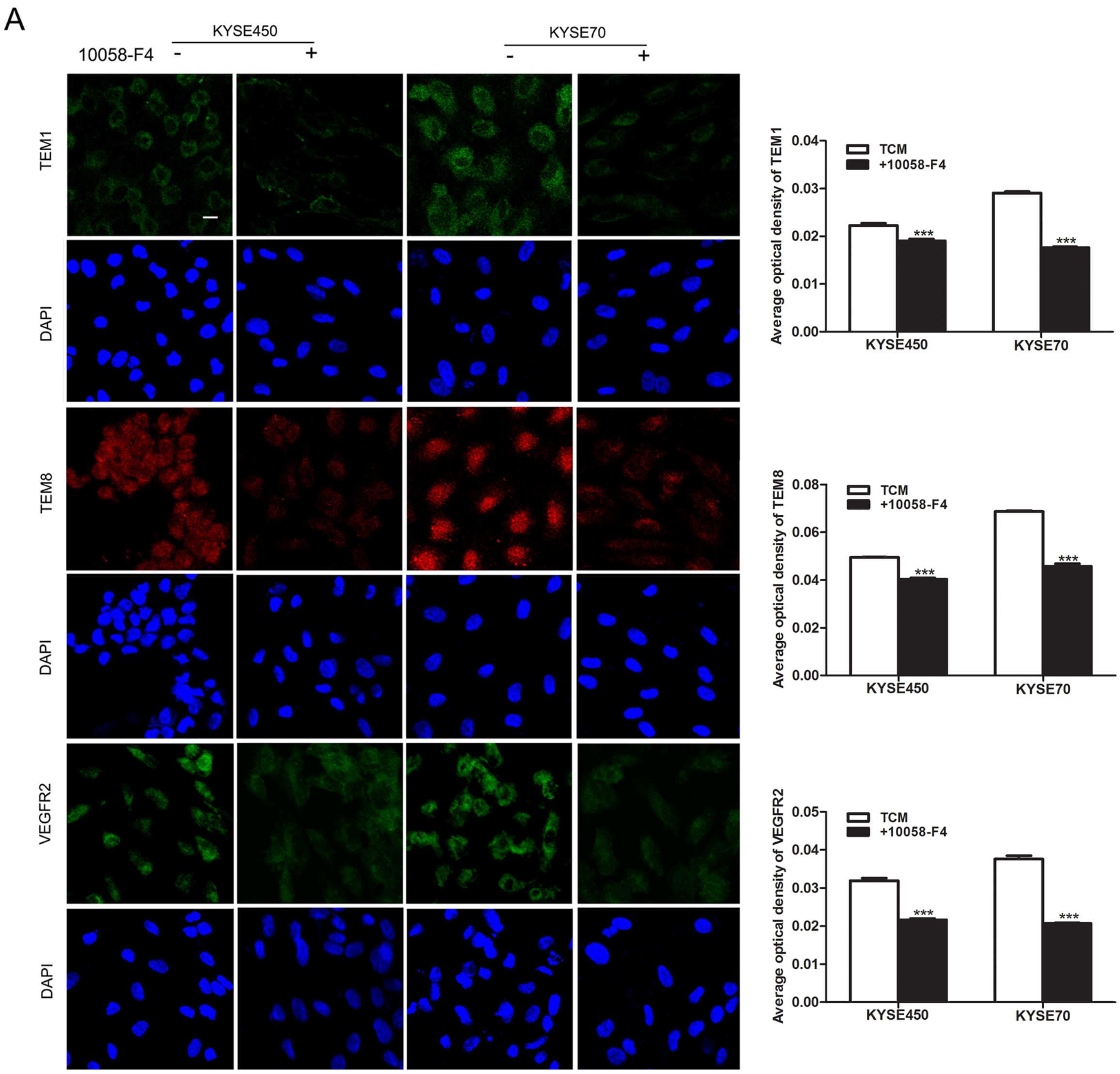

Supplementary Figure 3: 10058-F4 inhibited the transition of NECs toward TECs induced by KYSE450 or KYSE70 TCM. NECs were induced by KYSE450 or KYSE70 TCM with or without 10058-F4 for 48 h. The protein levels of TECs markers (TEM1, TEM8 and VEGFR2) were detected by immunofluoresence (scale bar 50μm). Data are shown as mean±SD. *** $p < 0.001$.

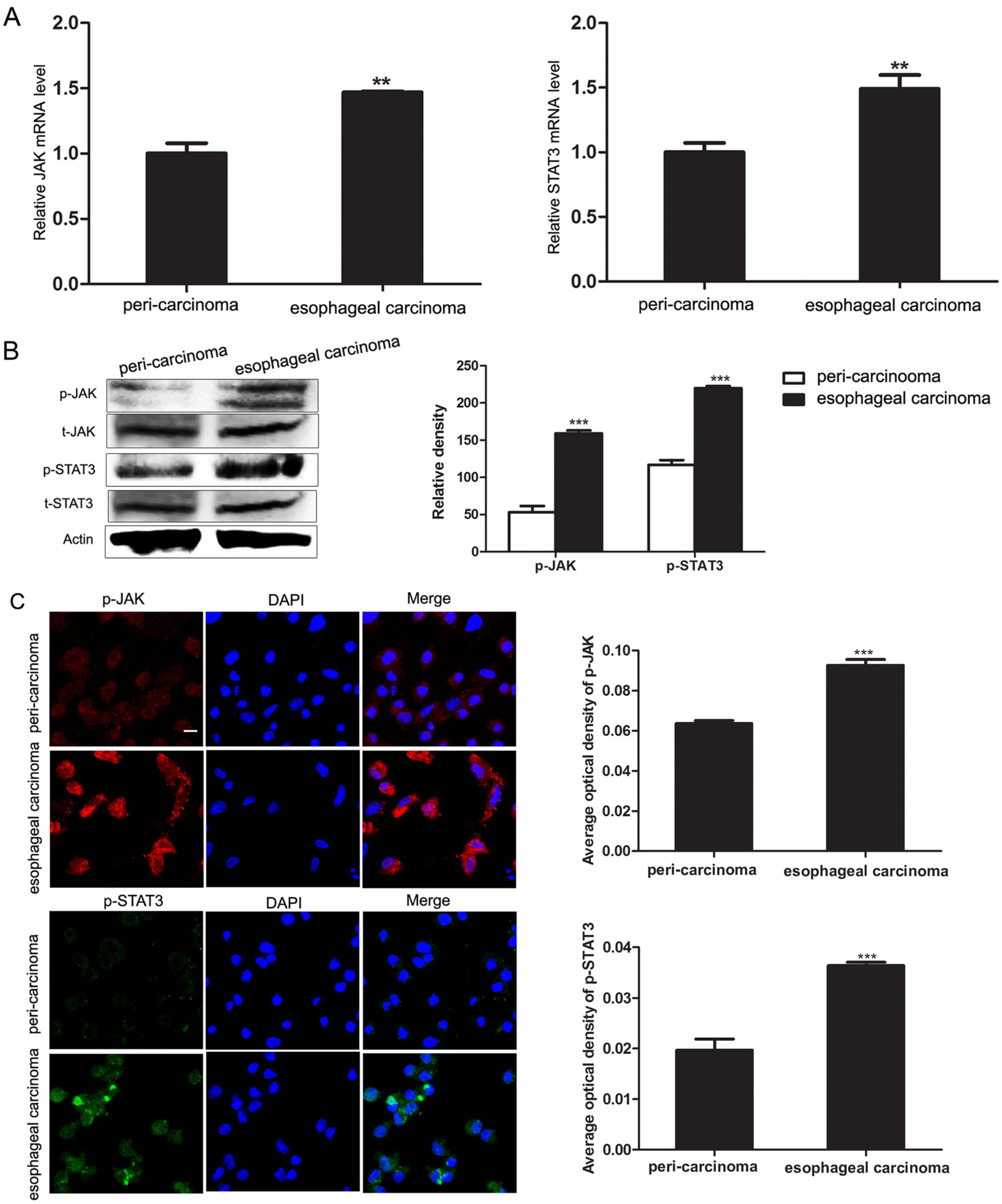

Supplementary Figure 4: JAK/STAT3 signaling pathway was activated during the transition of NECs towards TECs induced by ESCC tissue homogenate TCM. (A) NECs were induced by esophageal carcinoma or peri-carcinoma tissue homogenate TCM for 48 h. The relative mRNA levels of JAK and STAT3 were detected by qRT-PCR. **(B** and **C)** The expression levels of indicated proteins were detected by Western blot (B) and immunofluoresence (scale bar 50μm) (C). Results are expressed as mean±SD. ** $p < 0.01$, *** $p < 0.001$.

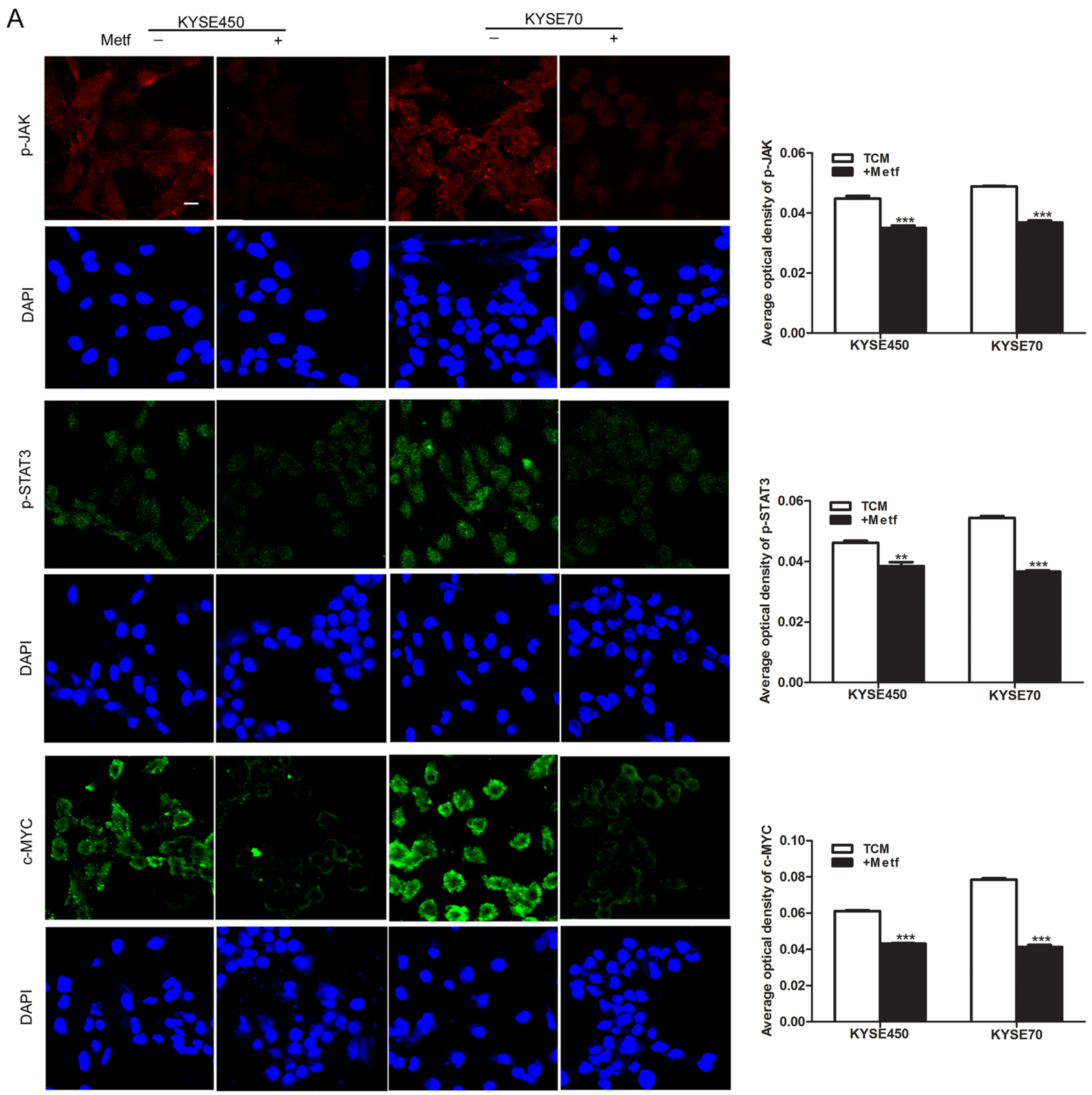

Supplementary Figure 5: Metformin suppressed the activation of JAK/STAT3/c-MYC signaling pathway. NECs were induced by KYSE450 or KYSE70 TCM with or without metformin for 48 h. The protein levels of p-JAK, p-STAT3 and c-MYC were detected by immunofluoresence (scale bar 50μm). Data are shown as mean±SD. ** $p < 0.01$, *** $p < 0.001$.

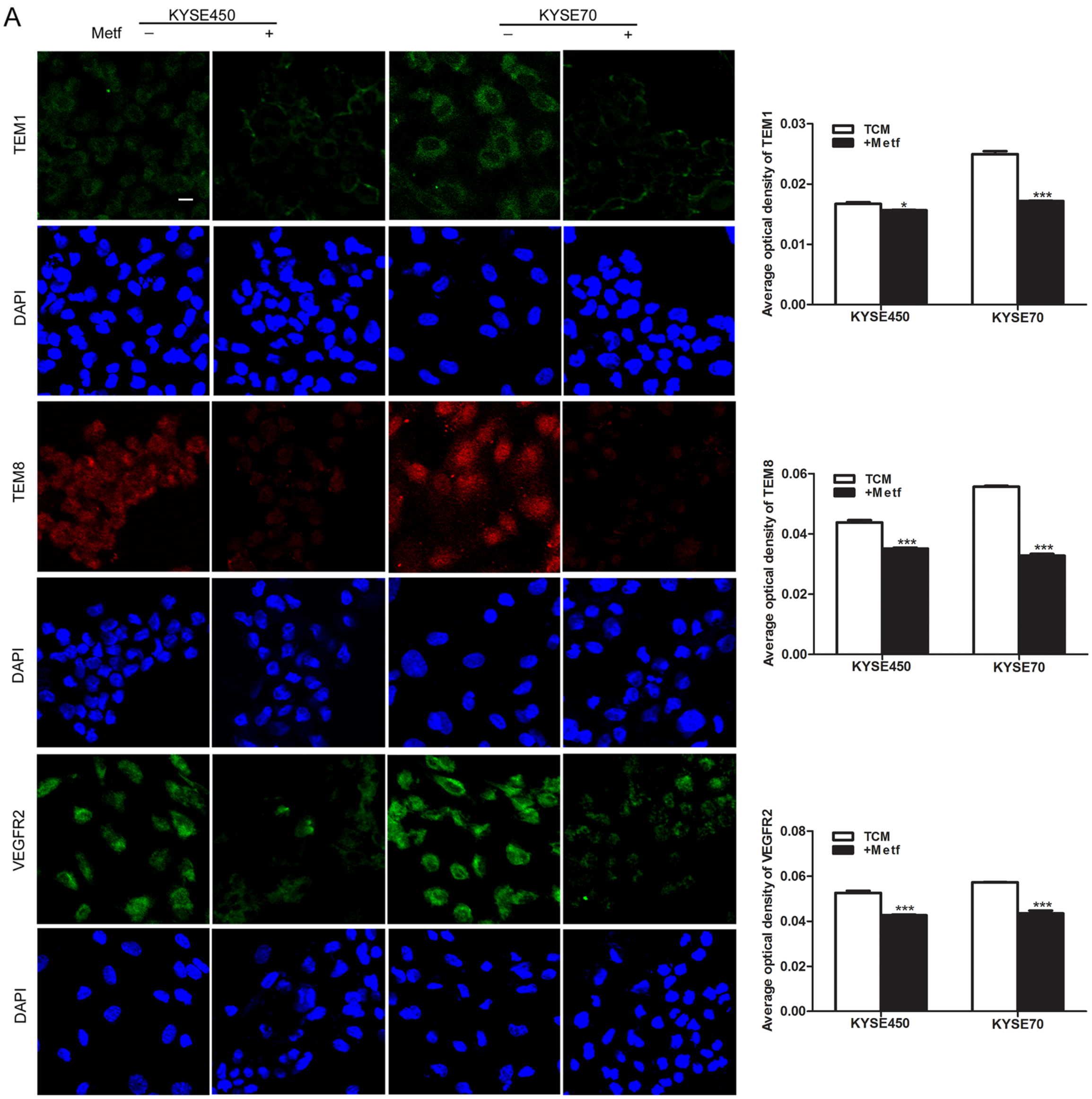

Supplementary Figure 6: Metformin inhibited KYSE450 or KYSE70 TCM-induced transition of NECs toward TECs. NECs were induced by KYSE450 or KYSE70 TCM with or without metformin for 48 h. The protein levels of TECs markers (TEM1, TEM8 and VEGFR2) were detected by immunofluoresence (scale bar 50μm). Data are shown as mean±SD. * $p < 0.05$, *** $p < 0.001$.
